# Supplementary material for: Orai1-mediated Ca2+ entry regulates lipolysis and mitochondrial activation in brown adipose thermogenesis
Source: Exp Mol Med. 2026 Aug 13;58(8):2781–91. doi: 10.1038/s12276-026-01808-x (PMC13538712; doi:10.1038/s12276-026-01808-x)
Supplement: Supplementary file 1 [file 12276_2026_1808_MOESM1_ESM.pdf]

Supplementary Figures

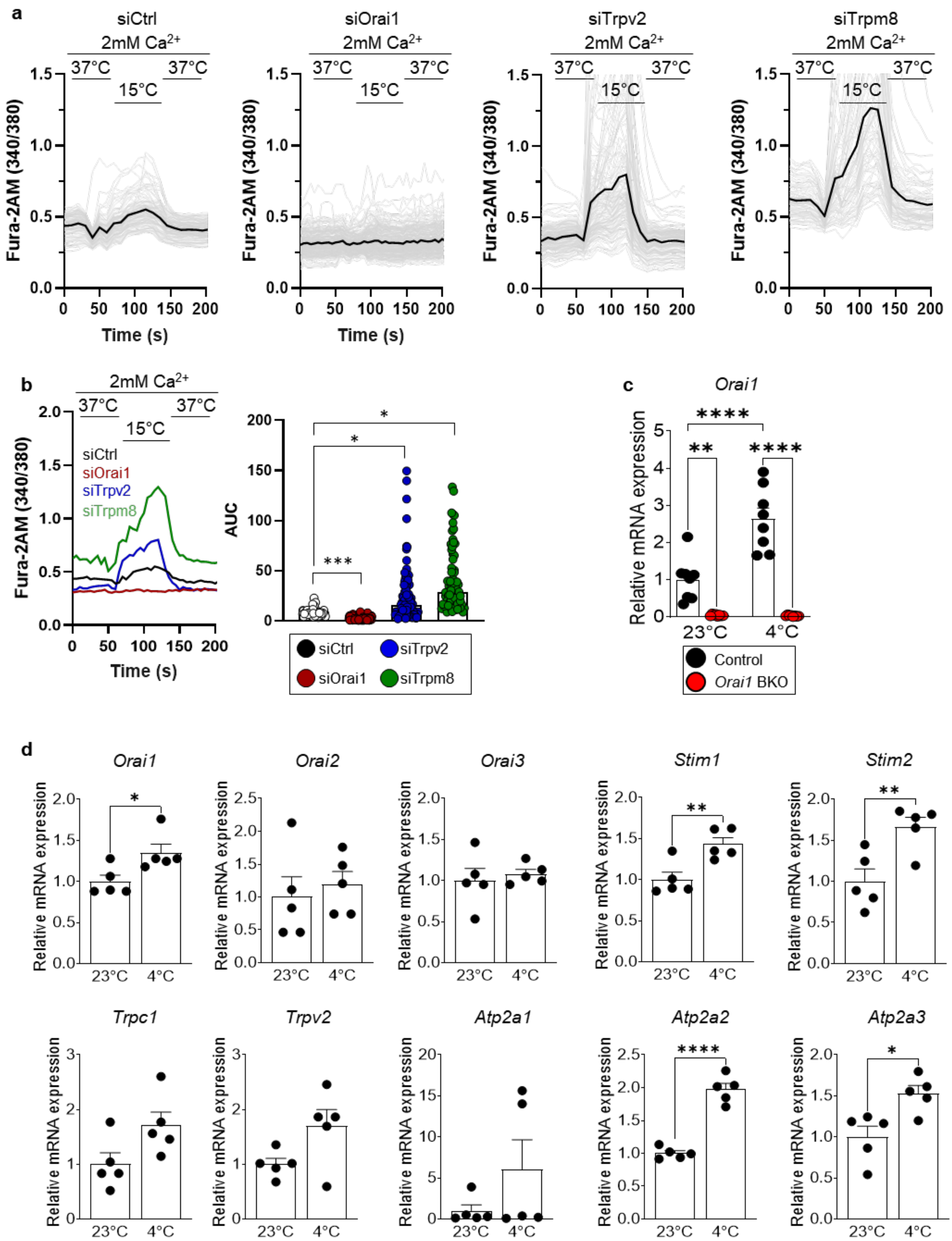

Supplementary Fig. 1 Orai1-related Ca^{2+} entry and its expression in BAT a Intracellular Ca^{2+} levels in primary brown adipocytes with *Orai1*, *Trpv2*, and *Trpm8* knockdown upon temperature shift from 37 °C to 15 °C. Black lines are average of each traces. **b** AUC analysis of **a**. **c** *Orai1* mRNA expression in BAT from control and Orai1 BKO mice exposed to acute cold exposure (23 °C or 4 °C for 6 h). **d** Expressional profiles of SOCE-related and Ca^{2+}-handling components under chronic cold exposure (3 days), obtained from the GSE70437 dataset. Data are presented as mean ± SEM. Statistical significance: *$p < 0.05$, **$p < 0.01$, ****$p < 0.0001$.

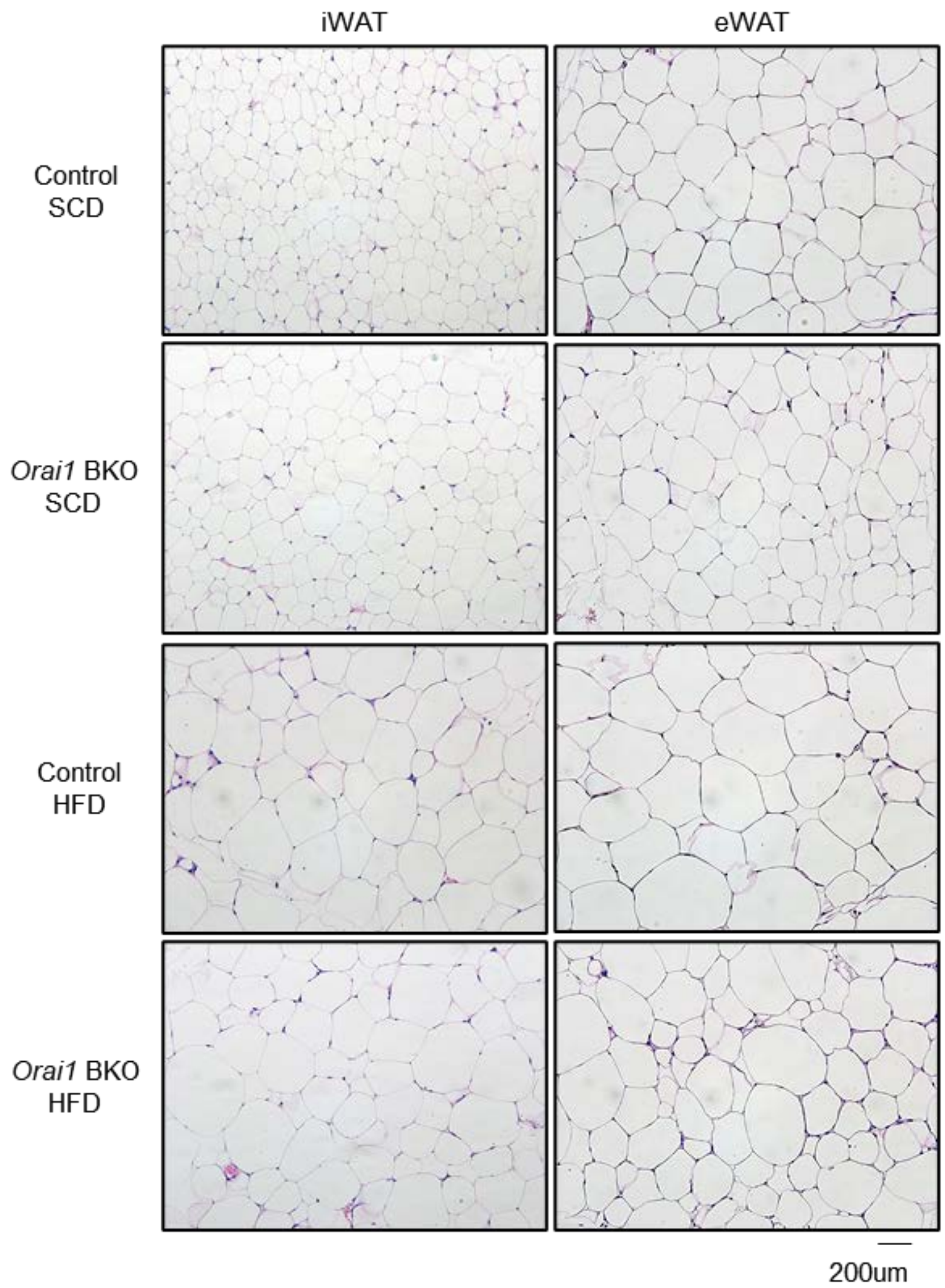

Supplementary Fig. 2 H&E staining of inguinal white adipose tissue (iWAT) and epididymal WAT (eWAT) from control and *Orai1* BKO mice fed with standard chow diet (SCD) or high fat diet (HFD)

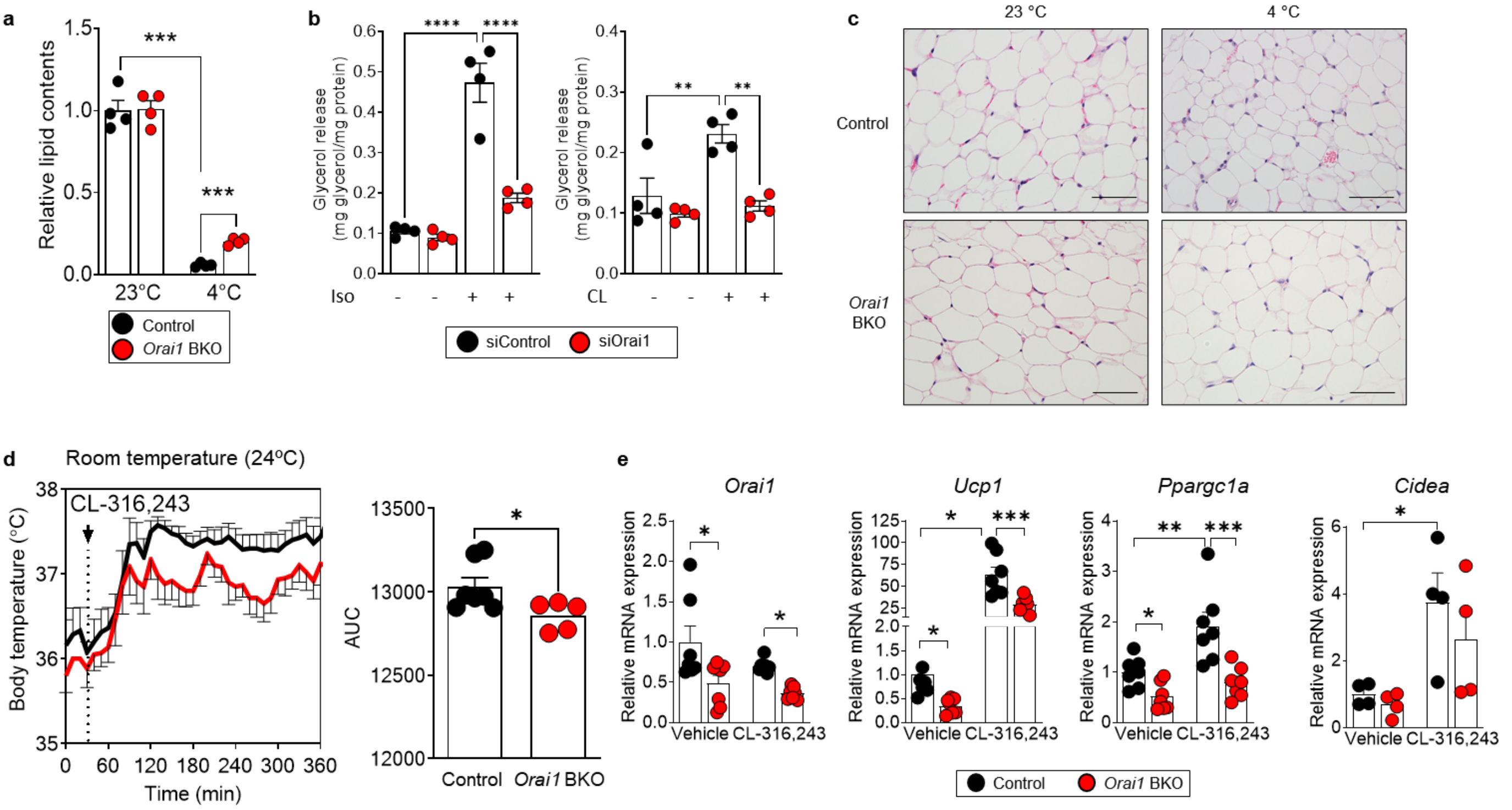

Supplementary Fig. 3 Lipolytic activation and thermogenic responses under cold and β-adrenergic stimulation

a Lipid contents of control and *Orai1* BKO BAT from H&E-stained sections (related to Figure 3e). **b** Glycerol release from brown adipocytes transfected with siCtrl or siOrai1 and stimulated with isoproterenol (Iso, 1 μM) or CL-316,243 (CL, 1 μM) for 2 h. Glycerol release was normalized to cellular protein content. **c** iWAT histology after 4 °C cold exposure (6 h). **d** Core temperature measured after CL-316,243 treatment (1 mg/kg i.p.) at room temperature. **e** mRNA expression of thermogenesis genes in primary brown adipocytes following CL-316,243 stimulation. Data are presented as mean ± SEM. Statistical significance: **p* < 0.05, ***p* < 0.01, ****p* < 0.001, *****p* < 0.0001.

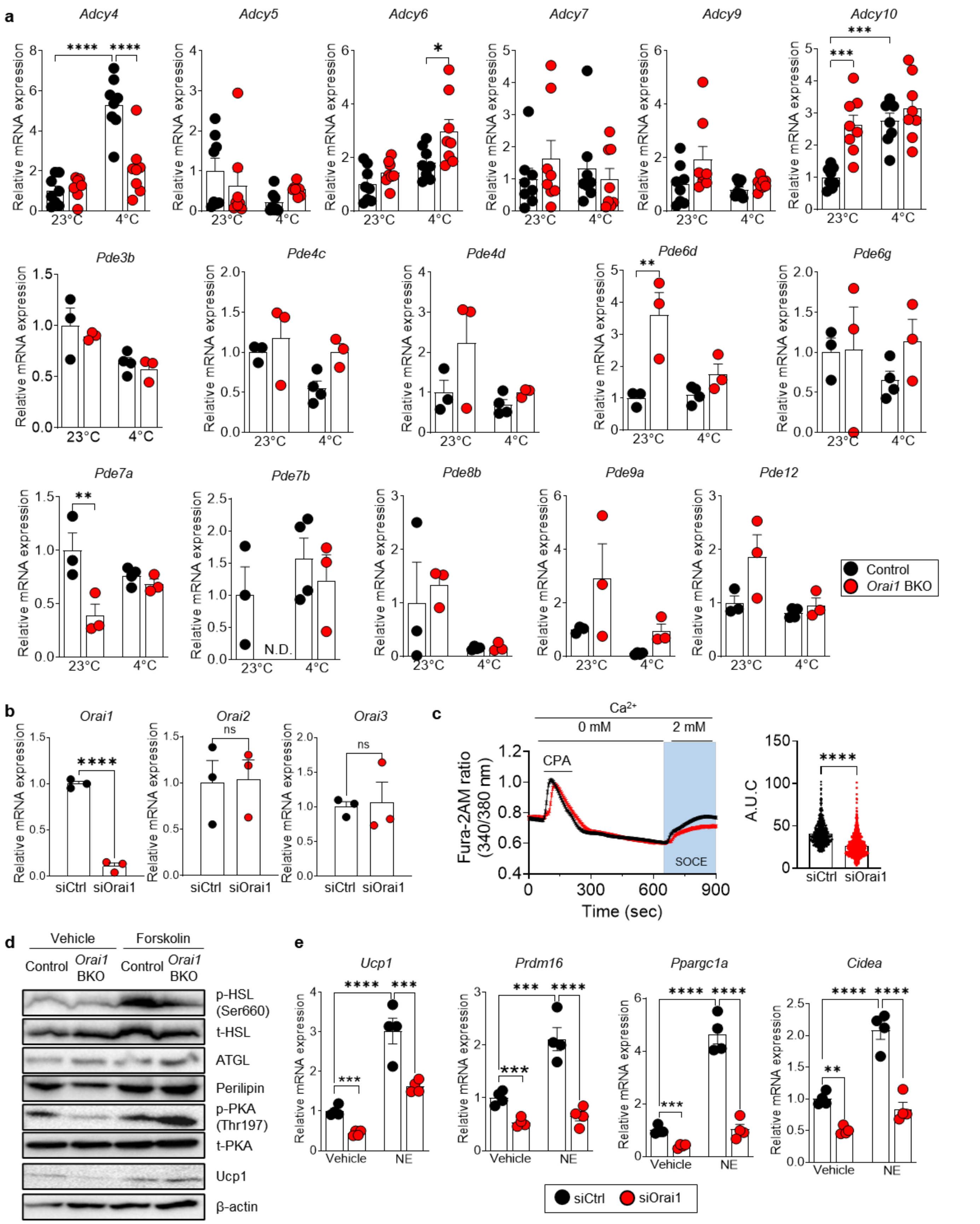

Supplementary Fig. 4 cAMP-related gene expression and signaling in *Orai1*-deficient brown adipocytes a mRNA levels of adenylyl cyclases and phosphodiesterases in BAT from control and *Orai1* BKO with cold exposure (6 h). **b** mRNA expression of *Orai1*, *Orai2*, and *Orai3* in brown adipocytes transfected with siCtrl or siOrai1. **c** Intracellular calcium levels measured using Fura-2 AM in brown adipocytes upon *Orai1* knockdown. CPA (20 μM) was applied as indicated. **d** Western blot of lipolysis and thermogenesis proteins in primary brown adipocytes from control and *Orai1* BKO with forskolin treatment. **e** mRNA expression of thermogenic genes from *Orai1* knockdowned brown adipocytes with norepinephrine (NE) treatment. Data are presented as mean ± SEM. Statistical significance: *$p < 0.05$, **$p < 0.01$, ***$p < 0.001$, ****$p < 0.0001$.

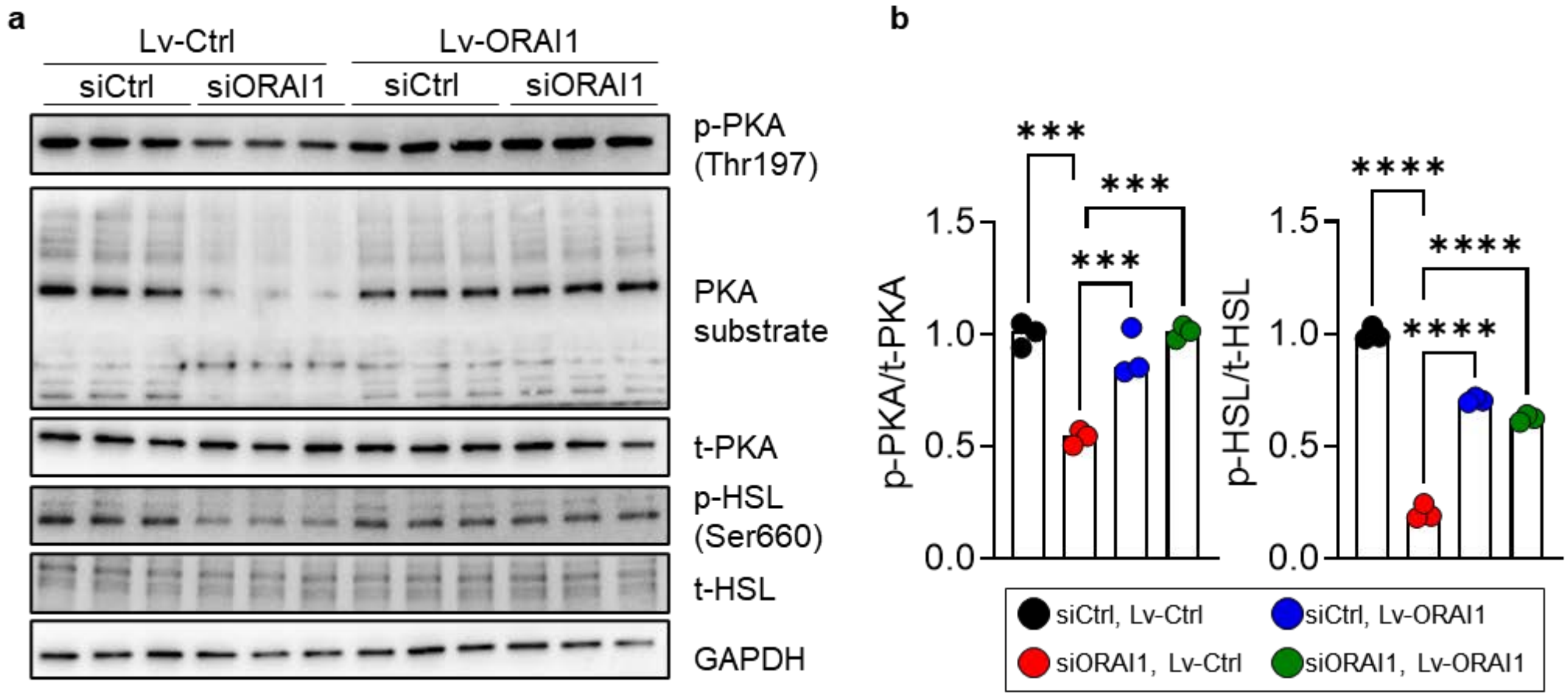

Supplementary Fig. 5 PKA signaling in human brown adipocytes following *ORAI1* knockdown and rescue a Western blot of PKA signaling and HSL phosphorylation under *ORAI1* knockdown followed by lentivirus-mediated *ORAI1* expression in human brown adipocytes. **b** Densitometric quantification from (**a**). Data are presented as mean ± SEM. Statistical significance: ***$p < 0.001$, ****$p < 0.0001$.

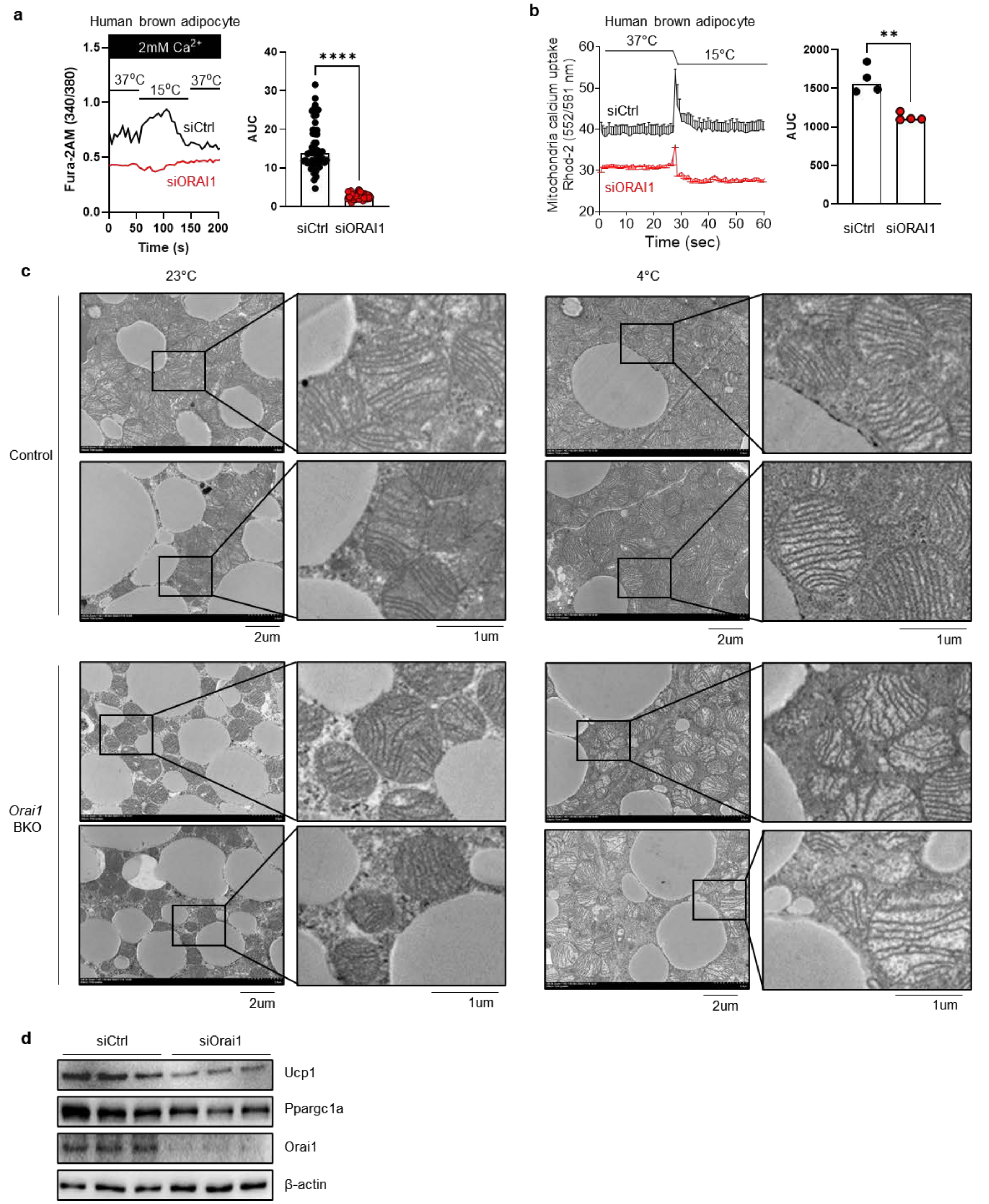

Supplementary Fig. 6 Mitochondrial Ca^{2+} dynamics and ultrastructure in *Orai1*-deficient brown adipocytes a, b Intracellular Ca^{2+} levels (**a**) and mitochondrial Ca^{2+} levels (**b**) in human primary brown adipocytes following temperature shift from 37 °C to 15 °C. **c** Transmission electron microscopy of BAT from control and *Orai1* BKO mice after 6 h cold exposure at 4 °C. Insets show magnified mitochondria. **d** Western blot of thermogenic proteins from brown adipocytes with *Orai1* knockdown.

Supplementary Tables

Supplementary Table 1. List of antibodies used in this study

Target protein	Source	Identifier
Orai1	Mybiosource	MBS3209798
Ucp1	Abcam	ab209483
Atgl	Cell Signaling	2138S
HSL (total)	Cell Signaling	4107S
HSL (p-Ser660)	Cell Signaling	45804S
Perilipin	Abcam	Ab3526
Pgc1a	Abcam	ab191838
PKA (total)	Cell Signaling	4782
PKA (p-Thr197)	Cell Signaling	4781
PKA substrates	Cell Signaling	9624S
OXPHOS Complex cocktail	Abcam	ab110413
b-Actin	SantaCruz	Sc47718
GAPDH	SantaCruz	Sc47724

Supplementary Table 2. Primer sequences used in this study

Gene name	Forward	Reverse
Ucp1	CTTTGCCTCACTCAGGATTGG	ACTGCCACACCTCCAGTCATT
Ppargc1a	GTCATTCGGGAGCTGGATGG	CAACCAGAGCAGCACACTCT
Prdm16	CACGGTGAAGCCATTCATATGCG	CTCCGATGCTTGTTGAGGG
Dio2	TTGGGGTAGGGAATGTTGGC	TCCGTTTCCTCTTTCCGGTG
Orai1	CCGGCTTCGCCATGGTAG	GGTGCAGGCACTAAAGACGA
Cidea	ATCACAACTGGCCTGGTTACG	TACTACCCGGTGTCCATTTCT
Orai2	GACACAGACGCTAGCCACGA	AGGATGGTTGACTGCCTGAC
Orai3	CCTTGGTGACTTGTGGTGGT	CTGCACAATCTCCCACTCGT
Rplp0	TCGTTGGAGTGACATCGTCT	TAGTTGGACTTCCAGGTCGC
Adcy3	AGTTTGTCATC GCCAGGGG	AAACACGCTACTCGGTGGAG
Adcy4	AGTACCCCTGGTCTGGAGTC	CTGGTGGCATCTTGTTCCCT
Adcy5	AGGACATTGGAGACAAGCTGTT	TACCATCTACACCCTGCTGC
Adcy6	TTGGCGATGGCAGAGATGAA	TGCTCCTGCGGTTCTTTCTT
Adcy7	CTTCGGGATGGAAAGCACCT	CTACGTGATGCCCAGGATGGC
Adcy9	AGAACACGGTGGTTGACAGAA	CAGCTACCAGGAAGAGGTCATA
Adcy10	GAACTGTCGGGGTTCTTCGT	CCTGCATCGCTGTCTGGTAT
Pde3b	AGGTTCCTGTAGGCCAAAGATTA	CTGCCACTCAATCCCTTGTGAA
Pde4c	GGATGGTCATAGACATGGTGCT	AGGCTGGTCACTTTCTTCGT
Pde4d	AGCCTGTCTTGTAAGCATTTCAG	TGCGGCTCTCAGTAGTTTGG
Pde6d	GACAATGCCTAGAAGGGGCA	CTCTCAAACATAGAAAAGCCTCAC
Pde6g	TTGGGTGGCTCCAGGTTCAT	AAGGGGTGAAAGGGTTTGGAG
Pde7a	ATATCAGTCGCCAGAACGAG	GCACATTTCAAGGCCATCTGTAG
Pde7b	ATGAACAAGCCGCACCG	GACTTCGCCACACCTCTCAA
Pde8b	AGGAAAGAGTGAAGGGAAGCC	GCGTTGCACAGGAAAGAGTT
Pde9a	CAGTATGGTCTGGCTCTGTG	GGGCATTGATCTGGTATGTATTG
Pde12	TCGGACCCACTGCACAAAG	GACTGAAGAACTGAAATCTGAAGG
Mt-Nd1	CCACGCTTCCGTTACGATCA	GTATGGTGGTACTCCCGCTG
Gapdh	GAACTCCTCATGGGTCTGTAGTG	TGTTGTGGTACGTGCATAGCTG
